# Freeform spectrometer enabling increased compactness

**DOI:** 10.1038/lsa.2017.26

**Published:** 2017-07-28

**Authors:** Jacob Reimers, Aaron Bauer, Kevin P Thompson, Jannick P Rolland

**Affiliations:** 1 The Institute of Optics, University of Rochester, Rochester, NY 14627, USA; 2 Synopsys, Inc., 3 Graywood Lane, Pittsford, NY 14534, USA

**Keywords:** aberrations, freeform optics, imaging spectrometer

## Abstract

We present optical designs with freeform optics in the context of hyperspectral imaging. Results show designs that are 5 × more compact in volume than similar designs using conventional spherical or aspherical surfaces. We will show how combining the concepts of spatial and spectral-band broadening, which will be introduced in this paper, led to the improvement in compactness that is uniquely enabled by freeform optics.

## Introduction

The need for compact, high-performance imaging spectrometers that span an application space from satellite imagery to food safety has never been higher. In this data-driven world, consumers want to know more about the chemicals in their food or how to precisely match the paint color on their living room wall. Companies need fast, reliable methods of product inspection with high spatial and spectral resolution, necessitating imaging spectrometer instrumentation. A pushbroom imaging spectrometer images a slit onto a 2D detector array, where the length (longest dimension) of the slit sets the full field of view (FOV) of the spectrometer and lies along one of the dimensions of the detector array, while the spectral dispersion created by the grating spreads along the orthogonal dimension, along which the width (smallest dimension) of the slit is imaged. An increase in system requirements for spectral range and resolution, compactness, and FOV of spectrometers has motivated the emergence of technologies and applications in hyperspectral imaging.

Wu *et al*^1^ used a series of lenses and a volume phase grating to diffract and image the spectral bandwidth. The transmissive volume phase grating allows the focal plane to be near the grating enabling a compact form. Using a refractive approach to the design of a hyperspectral imager resulted in a compromise between the spectral resolution and the spectral bandwidth over which the transmissive materials could operate, as refractive materials are intrinsically dispersive and unwanted dispersion over a broad spectral bandwidth must be corrected.

Bednarkiewicz *et al*^2^ introduced digital micromirror devices (DMDs) as a light modulator for excitation in fluorescence lifetime imaging. The DMDs direct various FOVs into a collection lens that feeds into a spectrometer (point-to-point). Combining all of the FOVs and the individual spectra constructs a hyperspectral image. With moving parts, this method is not targeted at snapshot or high-speed imaging of objects, as a 1024 × 768 pixel image with 15-nm spectral resolution requires over 4.5 h of image acquisition time.

Wang *et al*^3^ developed a design for cavity enhanced multispectral photodiodes. They used a traditional camera, but replaced the detector with one that is sensitive to varying wavelengths as a function of the depth of the pixel itself, using algorithms to compute the spectra at each point in the resulting image. The implementation of this approach resulted in spectral resolution and spectral bandwidth on the order of 50 nm and 1500-3500 nm, respectively.

As a precursor to the work presented in this paper, Bauer *et al*^4^ described how systems operating off-axis or in compact geometries enabled by tilted surfaces result in aberration contributions that are asymmetric in the FOV, which may negatively impact image quality if not mitigated. Bauer showed how these field-asymmetric aberrations may be corrected with freeform surfaces located both at and away from the aperture stop. Imaging spectrometers are inherently non-symmetric due to the dispersive element that separates the wavelengths and, thus, freeform surfaces may lift these systems to an unprecedented level of performance.

In the design concepts presented here, we are seeking formidable increases in resolution, imaging performance, and other system requirements including compactness and high-speed data collection in an all-reflective optical design where mirror surfaces are shaped as freeforms. We first discuss the theoretical background to establish a starting point for the spectrometer design together with a novel metric of performance analysis. We then present the design of two freeform spectrometers based on the Offner–Chrisp geometry; one using a freeform grating and the other using a hybrid spherical-freeform Mangin grating. Results show a 3 × increase in spectral bandwidth or a 2 × increase in slit length for the same volume as a non-freeform counterpart. Furthermore, results also show a 5 × reduction in volume when considering equal spectral bandwidth coverage, slit length, spectral resolution and imaging performance.

## Materials and methods

### Freeform surfaces

***Choice of freeform surface type.*** The so-called anamorphic optics involves optical designs with a combination of spherical, aspherical and toroidal surfaces—the last of which are the launching point for freeform optics. Freeform optics involve optical designs with at least one freeform optical surface where the freeform surface shape, according to the ISO standard 17450-1:2011, has no translational or rotational symmetry about axes normal to the mean plane and belongs to the class of complex invariance^5^. Mathematically, freeform surfaces may be defined as either global descriptions such as orthogonal polynomials^6, 7^, or local descriptions such as radial basis functions^8, 9, 10^ and non-uniform rational B-splines^11^. Local descriptions yield great challenges in optimization as a result of the large number of control parameters^12^, thus our focus will be on global descriptions. Historically, XY-polynomials were the first type of polynomials used for low-order freeform surfaces^13^. These polynomials remain a common surface description of freeform surfaces^14^, but their lack of orthogonality renders them less desirable than their orthogonal counterparts as the design complexity increases to require more than a few lower-order terms. The 2D Q-polynomials introduced by Forbes in 2012 (Ref 7) are orthogonal in gradient and aim to provide a freeform surface description where the root-mean-square (RMS) gradient of the normal departures from the best fit sphere may be readily computed from the sum of the squares of the polynomials’ coefficients. As such, the RMS gradient across the surface may be readily constrained in optimization resulting in an upper bound on the maximum surface slope that will facilitate fabrication or associated optical metrology during manufacturing of the surface. The full impact that 2D Q-polynomials have on a design, both for manufacturing and performance, is under investigation^15^. Zernike polynomials are a global description that are orthogonal in sag over a unit disk and are widely used in optical fabrication and testing. They come in various orderings and normalizations, but the FRINGE Zernike set has been adopted as an industry standard for surface descriptions in those fields.

***Design with freeform Zernike surfaces.*** In the work presented here, the FRINGE Zernike set is used as it follows the ordering of the traditional Seidel aberrations^16^, which is preferred by optical designers since third-order aberrations are listed before the fifth-order aberrations. They will be referred to as Zernike polynomials hereafter. According to the ISO standard 10110-19, the sag, z, of a Zernike freeform surface is mathematically represented by


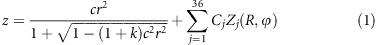


where *c* is the curvature of the base sphere, *k* is the conic constant, *r* is the radial coordinate of the surface, *R* is the radial coordinate normalized by *R*_*norm*_, that is *R*=*r/R*_*norm*_, *φ* is the azimuthal component of the aperture, and *C*_*j*_ is the weight factor of the *j*th Zernike term, *Z*_*j*_^17^. In design, a surface is considered freeform when it involves at least a Zernike coma term of some order (that is, where a variation of sag around the aperture is an odd number of cycles) that may be tailored independently of other Zernike terms. This definition highlights that, in design, freeform surfaces go beyond anamorphic surfaces as defined earlier but also off-axis conic sections of otherwise rotationally symmetric surfaces as off-axis conic sections involve coma but in a fixed relationship to other Zernike terms such as spherical aberration and astigmatism.

An important aspect of Zernike polynomials that make them suited for design is that they display a direct connection to optical aberration theory^18^. Specifically, the aberration contribution of each Zernike polynomial term that defines the full shape of a surface either at or away from the aperture stop can be expressed mathematically as a function of field. As an example, a freeform surface shape described by Zernike coma on a surface away from the aperture stop contributes a wavefront aberration, *W*_NotStop_, expressed as


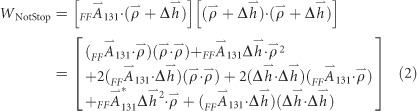


where 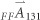 is a two-dimensional vector that describes the magnitude and orientation of the Zernike coma overlay, 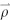 is the normalized pupil vector, and 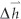 is a field-dependent term that arises when the surface is located away from the stop. The first term represents field-constant coma, the second term is field-asymmetric, field-linear astigmatism, the third term is field-linear medial field curvature, the fourth term is a field-quadratic distortion, the fifth term is an anamorphic field-quadratic distortion and the sixth term is piston. The distortion and piston terms do not affect the image quality but, rather, the mapping and phase, respectively. Understanding the field dependence of the aberrations created by each Zernike overlay type on a surface away from the aperture stop is essential to designing freeform optical systems.

Importantly, we recommend as a best practice in design to constrain *Z*1 and *Z*2/*Z*3 (piston and *X/Y* wedge, respectively) in the optimization to be nonzero such that the net sag and slope at the vertex are identically zero. As an example, consider the net sag and the *Z*9 term, or Zernike spherical aberration. *Z*9 is given as


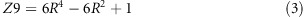


where the first, second and third terms are a quartic dependence in the pupil (that is, spherical aberration), a quadratic dependence in the pupil (that is, defocus) and a constant (that is, piston). The coefficient to the *Z*1 component on this surface must be nonzero and, in fact, must be equal and opposite to the coefficient on the *Z*9 component in the absence of any other terms on the optical surface so the sum of the piston contributions is zero. Similarly, the surface tilt at the center of any freeform surface is constrained to zero, which typically involves non-zero coefficients for the *Z*2/*Z*3 Zernike terms to offset equivalent terms in coma related Zernike terms. When appropriately constrained, a freeform surface will require no translation or tilt in the Zernike representation and the optical layout will correspond to the mechanical blueprint.

### Performance evaluation of spectrometers

**Figure 1:**
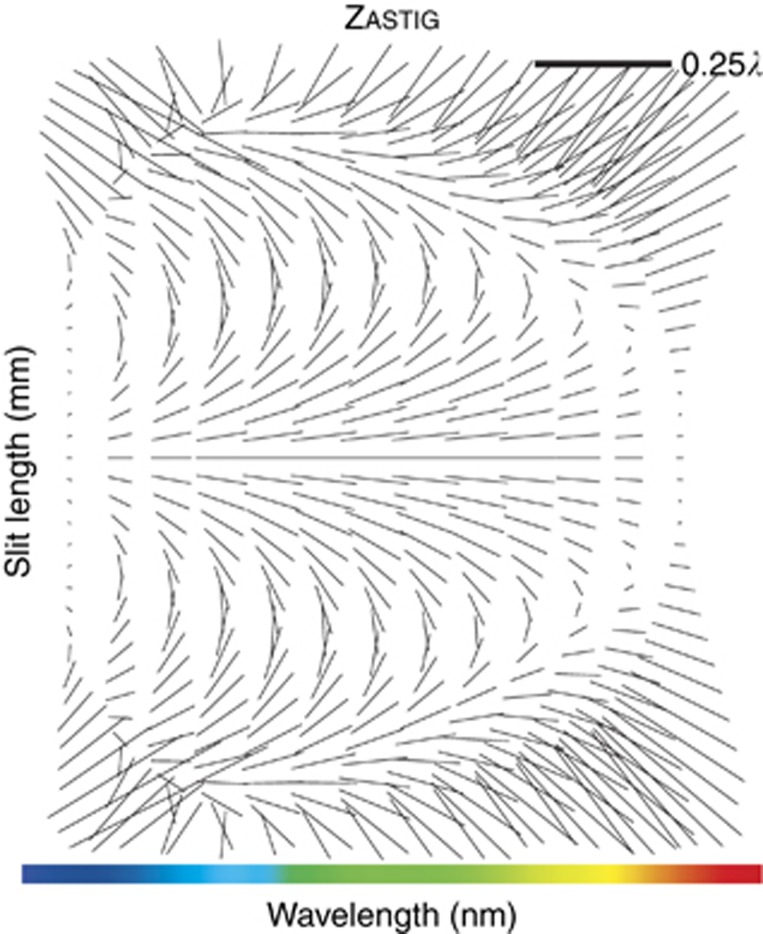
Representative 2D SFFD for Zernike astigmatism in an imaging spectrometer. The vertical axis is the slit length. The horizontal axis is the spectral bandwidth that contributes together with the dispersion element at creating a FOV component for the tertiary mirror that is perpendicular to that of the slit.

***Spectral full-field display.*** We first introduced the concept of the spectral full-field display (SFFD) at the 2014 International Optical Design Conference^19, 20^. An example of the novel display, which builds on the conventional full-field display, is shown in Figure 1. An SFFD plots the magnitude and orientation of a Zernike aberration component of the wavefront over a grid of points that sample the slit length along the vertical axis, and the spectral bandwidth along the horizontal axis. The SFFD enables rapid visualization of the aberrations and performance (that is, RMS wavefront error) of an imaging spectrometer and guides the design process, particularly when implementing freeform surfaces into the spectrometer. The magnitude of the aberrations is expressed in waves, where the zero to peak magnitudes of the aberrations computed in microns are divided by the local wavelength, which allows for wavelength-specific performance analysis. The SFFD is used throughout this research to iteratively design, quantitatively evaluate and visually compare different classes of designs.

***Distortion.*** Distortion is an imperative metric for imaging spectrometers—often the optical designer must make a compromise between achieving high optical performance and low distortion. In an imaging spectrometer, the two main types of distortion are spectral smile and spatial keystone^21^. Spectral smile is calculated at each wavelength as the maximum deviation from the average *x*-centroid position for each point along the slit. Spatial keystone is calculated at each point along the slit as the maximum deviation from the average *y*-centroid position for each wavelength. Both types are addressed in this work.

### Offner and Offner–Chrisp concepts

**Figure 2:**
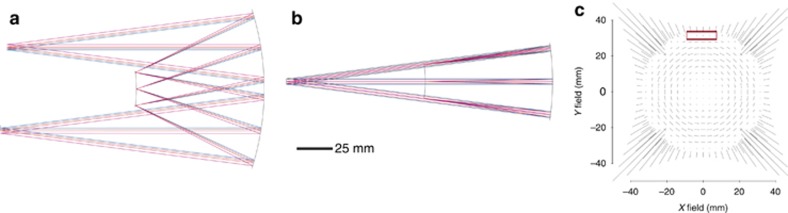
Layout of a ring-field Offner 1:1 relay. The plane perpendicular to the slit length is shown in (**a**), the top view of **a** is shown in (**b**) in the plane of the slit length and the ring-field correction is shown in (**c**), where the central part of the field is dominated by third-order astigmatism and the edge of the field shown is dominated by fifth-order astigmatism. The region outlined in red is where third- and fifth-order astigmatism balance and is called the ring field zone of correction.

One prolific all-reflective design for imaging spectrometers is based on the Offner ring-field geometry. Illustrated in Figure 2a and 2b, the most classical Offner geometry is a 1:1 relay consisting of two quasi-concentric spherical mirrors, where the primary mirror is used twice in reflection^22, 23^. This design form is corrected for spherical aberration as the parent mirrors operate near their common center of curvature. The design is also corrected for coma by symmetry about the stop. Finally, the Offner relay is corrected for astigmatism when operating within the ring-field zone where third- and fifth-order astigmatism balance, as shown in Figure 2c, where the sagittal and tangential focus lines touch at the edge of the FOV.

The Offner relay geometry was first adapted to spectrometers by Thevenon and Mertz^24^, detailed by Kwo^25^ and later by Lobb^26^ in 1987 and 1994, respectively, and it gained the name of the Offner spectrometer. In the Offner spectrometer, the secondary mirror of the Offner relay is replaced by a reflective, convex grating whose groove spacing is chosen based on the required spectral dispersion and physical dimensions of the focal plane, which combined with a pixel size aim at a targeted spectral resolution. As with the classical Offner relay, the Offner spectrometer operates at the ring-field balance of astigmatism, but only for a single wavelength, which is typically chosen to be the central wavelength of the spectral bandwidth.

In 1999, Chrisp developed a modified geometry for an imaging spectrometer, which builds on the understanding that the diffraction grating on the secondary mirror breaks the symmetry of the traditional Offner^27, 28^. As a consequence, a split of the primary mirror into two mirrors with different radii, while retaining the near-concentric property of the classical Offner relay, provides additional degrees of freedom toward the correction of aberrations. The radius of curvature of the convex grating in the Offner–Chrisp imaging spectrometer, *R*_g_, is governed by the grating density, *l*, and the spectral dispersion at the image plane, ζ, where *m* is the grating order and may be calculated as^26^


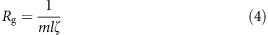


Since the three optical surfaces are concentric, Equation (4) combined with the distances between the grating and the primary and tertiary mirrors may be used to estimate the size of the spectrometer. Specifications such as spatial and spectral image size determine a choice of *R*_g_ after which the radii of curvature of surfaces 1 and 3, *R*_1_ and *R*_3_, may be iteratively computed^28^.

## Results and discussion

### Benchmarking of an Offner–Chrisp imaging spectrometer

**Figure 3:**
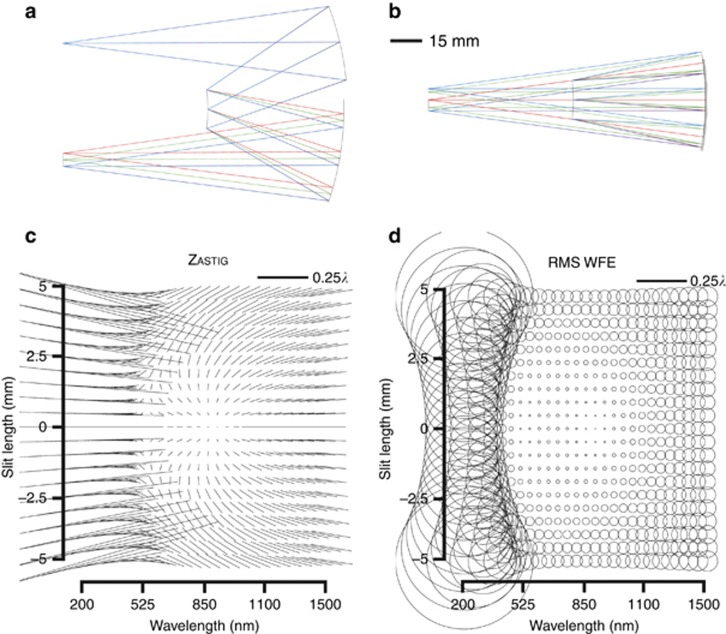
Offner–Chrisp imaging spectrometer baseline design with all-spherical surfaces, where *R*_1_=−130.9 mm, *R*_g_=−66.7 mm, *R*_3_=-129.2 mm. (**a**) The plane of spectral dispersion, (**b**) the plane of the slit length. SFFD showing both (**c**) astigmatism and (**d**) RMS WFE.

The Offner–Chrisp imaging spectrometer geometry is shown in Figure 3 as a geometry of example to show the freeform advantage. Results are expected to broadly apply to various geometries^29, 30^. The Offner–Chrisp is a three mirror, ring-field design with 1:1 magnification where all three mirrors are concentric, with a diffraction grating placed on the convex secondary mirror surface^27, 28^. To guide the discussion, we specify an F/3.8 Offner–Chrisp imaging spectrometer geometry spanning 200–1500 nm in spectral bandwidth. The slit length to the spectrometer is 10 mm (out of plane) and the spectral dispersion is 100 nm mm^−1^. The primary and tertiary mirrors have different radii of curvature as labeled in Figure 3. Using Equation (4), with *m*=1 and a grating density of 150 lines per mm, the grating radius of curvature was calculated to be −66 mm. The primary mirror sees a 1D full FOV set by the slit length that is aligned to the ring-field balance zone. The slit in the designs has a finite width equal to the pixel size (that is, 10 μm) and is fully inside the ring-field, thus the optical performance over the width of the slit is quasi-constant requiring only a line slit to be modeled in the comparison of the various systems. The tertiary mirror, however, sees an asymmetric 2D full FOV, where the 1D full FOV set by the slit length (that is, spatial FOV) is seen by the tertiary after being imaged through the primary mirror and the grating and in the orthogonal dimension the tertiary sees an angular spectral bandwidth, *θ*_*W*_, that may be thought of as a spectral FOV created by the grating. Recognizing the existence of spatial and spectral FOVs seen by the tertiary mirror is central to developing a strategy to correct the optical aberrations of an imaging spectrometer and to capturing the correction with the SFFD. By design, the astigmatism is minimized for the central wavelength using the ring-field zone property in the Offner geometry. Upon analysis, the variation of astigmatism with wavelength is found to be the limiting aberration, thus the SFFD for Zernike astigmatism will be presented alongside the RMS wavefront error (WFE) in performance analyses.

***All-spherical design.*** The Offner–Chrisp imaging spectrometer design form, composed of all-spherical surfaces, is often used in state-of-the-art imaging spectrometers for both military and civilian aerospace applications^31, 32^. The all-spherical performance is shown in Figure 3c and 3d and is used as a baseline comparison for the purpose of highlighting differences between non-spherical surface types including freeform surfaces. Astigmatism limits on-axis performance in the spectral bandwidth from 200 to 500 nm and also experiences further deterioration along the slit length.

**Figure 4:**
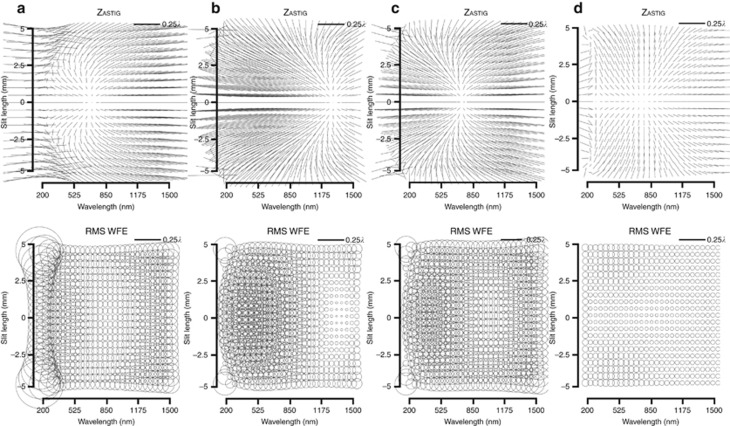
SFFDs for variously shaped surfaces. SFFD showing both astigmatism and RMS WFE for (**a**) all-aspheres, (**b**) all-aspheres with astigmatic nodal shift targeted in optimization, (**c**) all-biconic aspheres, (**d**) freeform surfaces, where smaller symbols represent better performance.

***All-aspherical design.*** In order to fully investigate the freeform advantage, it is essential to understand whether using all aspherical surfaces will help improve the performance of the all-spherical design. Two different aspherical formulations are compared. Figure 4a shows the resulting performance when allowing the fourth- and sixth- order aspherical coefficients denoted as A and B, respectively on each of the surfaces. Additional coefficients in optimization did not help the performance. Note that the RMS WFE is still dominated by the performance in the ultraviolet region due to the scaling of the local wavelength, which prompts the optical designer to look at the dominant aberration using the SFFD and work on correcting it in that region. In Figure 4b, the astigmatic nodal region was moved in optimization by varying the same aspherical coefficients (A–B) together with weighting the lower wavelengths in proportion to the relative magnitude of the astigmatic aberration in that spectral region. Results show an improved astigmatic field in that region and a decrease in the maximum RMS WFE by over 50%. This corrective technique is akin to changing the wavelength where the astigmatism is minimized in the first-order design, with the added benefit of retaining the first-order properties and packaging without redesigning the entire system. Results also show, however, that the average RMS WFE does not decrease in proportion and, in fact, increases slightly from 0.107*λ* to 0.116*λ* as shown in Table 1, which led us to next investigate the use of anamorphic surfaces, specifically biconic aspheres. Figure 4c shows the performance when all the optical surfaces have been extended to biconic aspheres and optimized for the two conic constants and the aspherical coefficients (A–B). Adding more coefficients to the aspheres did not improve optical performance. Results show that the maximum RMS WFE decreased by a factor of 4 compared to the all-spherical design, while the average RMS WFE, in fact, increased by 9% as reported in Table 1.

***Freeform design.*** The next step was to introduce freeform surfaces in the optical design using strategic low-order (up to 21 terms) FRINGE Zernike polynomial terms for each of the two mirrors as well as the diffraction grating. The terms are added incrementally by first displaying the aberrations of the system using SFFDs and then determining the limiting aberration. The Zernike freeform term that would best correct the limiting aberration can be determined from the freeform aberration theory described herein and then added to a surface either at or away from the stop. The weighting of that term is then optimized after which the process is repeated until no further correction is required. The freeform spectrometer performance is shown in Figure 4d and is diffraction limited over the entire slit length and spectral bandwidth. While the conventional aspheres or biconic aspheres first presented did not appreciably decrease the average RMS WFE over the specified performance space, the use of freeform surfaces resulted in an average RMS WFE decrease of 65%. A quantitative comparison of the approaches is presented in Table 1.

### Freeform imaging spectrometer advantages

**Figure 5:**
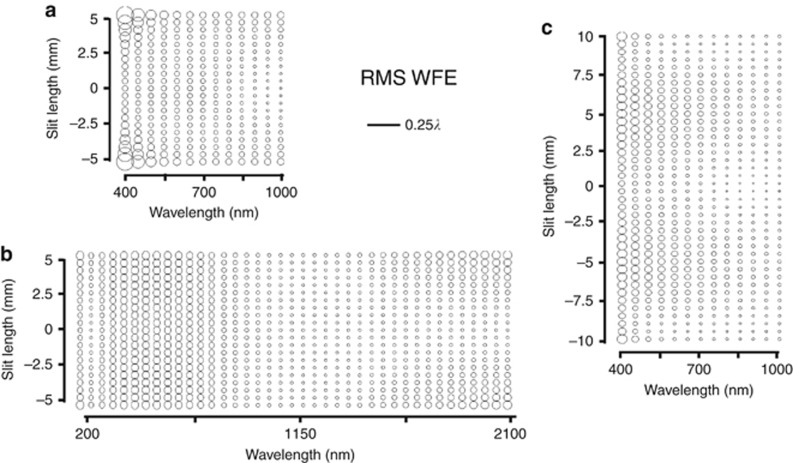
Spectral-band and spatial broadening of an F/3.8 Offner–Chrisp imaging spectrometer. SFFD showing RMS WFE for the (**a**) all-spherical baseline design, (**b**) freeform spectral-band-broadened performance, where the spectral bandwidth increased 3.1 × from 600 nm in the all-spherical design to 1900 nm in the freeform imaging spectrometer and (**c**) freeform spatial-broadened performance, where the slit length increased 2 × from 10 mm in the all-spherical design to 20 mm in the freeform imaging spectrometer.

***Spectral-band broadening.*** In the previous section, it was shown that freeform surfaces can offer aberration correction in both the spatial and spectral dimensions at the image plane. Here we isolate the spectral-band broadening in the system by holding the slit length constant and increasing only the spectral bandwidth. To highlight the benefit of freeform over the all-spherical baseline design, the astigmatism was minimized at 700 nm and the system specifications of spectral bandwidth and slit length for the all-spherical design were pushed to the maximum limit for which all wavelengths and fields were diffraction limited (<0.07 waves RMS WFE). The resulting nominal spectral bandwidth for the all-spherical design was 400–1000 nm as shown in Figure 5a. All three surfaces in the imaging spectrometer were selected to be freeform and the spectral bandwidth was increased until a field point for any wavelength failed to be diffraction limited. This optimization was also repeated for varying numerical aperture values. Each time, the freeform designs showed at least a 3 × increase in the spectral bandwidth for a diffraction limited system over the all-spherical design. We call this increase in allowable spectral bandwidth spectral-band broadening. The resulting spectral-band broadening is shown for an F/3.8 system in Figure 5b.

***Spatial broadening.*** In a separate simulation, the increase in slit length was investigated by holding the spectral bandwidth fixed at 400–1000 nm while the slit length was increased incrementally. The three surfaces were made to be freeform and the slit length was increased until a field point for any wavelength failed to be diffraction limited. This optimization was also repeated for varying NA and each time the freeform designs showed at least a 2 × increase in the slit length for a diffraction limited system over the all-spherical design. This increase in slit length is referred to as spatial broadening. The resulting spatial broadening is shown for an F/3.8 system in Figure 5c.

While in themselves, these two concepts–spectral-band and spatial broadening in a spectrometer design–enable increased spectral bandwidth and slit length, they will also reveal to be central to the understanding of how freeform optics enables correcting aberrations in a significantly more compact design.

***Increased compactness.*** One promise of freeform optics is the ability to create more compact designs compared to non-freeform prior art. Here we report on significantly decreasing the package size, as enabled by freeform surfaces^33^.

**Figure 6:**
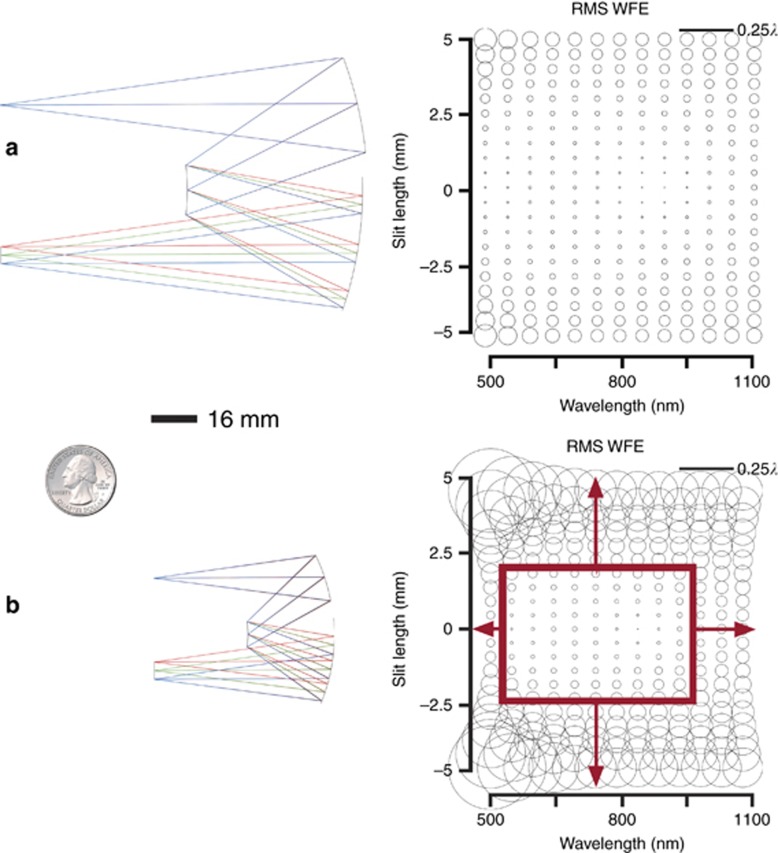
Offner-Chrisp baseline versus compact all-spherical designs and associated performances. (**a**) Offner–Chrisp baseline all-spherical design. The volume is 530 cm^3^. SFFD showing the RMS WFE. (**b**) Offner–Chrisp compact all-spherical design. The volume is 100 cm^3^. SFFD showing RMS WFE. The region contained in red is diffraction limited, and the desired performance bounds are indicated by the arrows necessitating both spatial and spectral-band broadening. *R*_1_=−63.8 mm, *R*_g_=−33.3 mm, *R*_3_=−64.1 mm.

As a way of showing the benefit of freeform surfaces, we leverage the previously shown increase in spatial and spectral-band broadening for freeform Offner–Chrisp imaging spectrometers and use this capability combined with the angular spectral bandwidth of the grating, *θ*_*W*_. The starting point to the design remains an F/3.8 Offner–Chrisp imaging spectrometer geometry, but in this case the wavelength spans 500−1100 nm as this range yields a diffraction limited design over the full FOV and spectral bandwidth. As in prior designs, the slit length is 10 mm and the spectral dispersion is 100 nm mm^−1^. The limiting aberration is astigmatism, which is minimized as in the Offner configuration for the central wavelength. We nominally used a 150 lines per mm diffraction grating. The groove spacing on the diffraction grating was chosen based on the spectral bandwidth, the spectral dispersion at the image plane, and the physical dimension of the focal plane. The diffraction grating creates the angular spectral bandwidth and these ‘spectral fields’ follow the imaging equation *y*=*f* tan(*θ*), where *y* is the height of a ray mapped onto the detector, *f* is the focal length of the optics between the grating and the detector, and *θ* is the angle of a diffracted ray with respect to the central wavelength. By considering all the diffracted rays, a range of FOV is created for the optics that follows the grating. By changing the diffraction grating to be more dispersive, we must also change the focal length, *f*, to image equivalently both spatially and spectrally on the focal plane. This solution results in a volume of 530 cm^3^ and the optical performance is given in Figure 6a. The system is reasonably diffraction limited over the slit length and spectral bandwidth.

In order to decrease the physical size of the spectrometer, we made the equivalent focal length shorter, which required a decrease in the groove spacing to retain the spectral dispersion at the focal plane. Specifically, with the objective to decrease the package size by at least 2 ×, we made the grating more dispersive by doubling the groove density (that is, 300 lines per mm) and scaled the focal length to 33 mm to maintain focal plane dimensions. In this new configuration, the field bias was changed in order to retain the ring-field balance between the third- and fifth-order astigmatism. This approach and solution resulted in a volume of 100 cm^3^, a decrease of 5 × in volume. The optical performance of this new starting point for optimization is given in Figure 6b. Simply scaling down the system yields performance that is far from diffraction limited for the desired slit length and spectral bandwidth. Decreasing the effective focal length led to using the mirrors at a much faster *F-*number than in the nominal system, which yield larger surface aberration contributions as shown in Figure 6b, particularly from field-dependent aberrations such as the limiting aberration, astigmatism.

**Figure 7:**
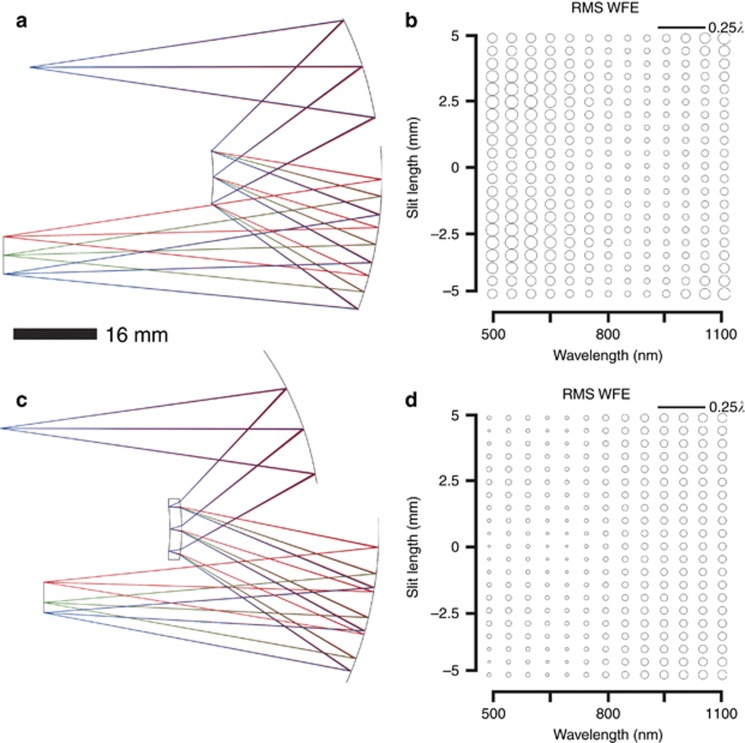
Compact freeform designs and associated performance. (**a**) Offner–Chrisp compact freeform design. The volume is 100 cm^3^. (**b**) SFFD showing RMS WFE for the compact freeform performance. *R*_1_=−62.4 mm, *R*_g_=−34.2 mm, *R*_3_=−67.6 mm. (**c**) Offner–Chrisp compact freeform Mangin design. The volume is 100 cm^3^. (**d**) SFFD showing RMS WFE for the compact freeform Mangin performance. The performance is diffraction limited over the full FOV for the spectrum in both cases. *R*_1_=−57.1 mm, *R*_g_=−34.5 mm, *R*_3_=−69.9 mm.

As exemplified in Figure 5, freeform surfaces in an Offner–Chrisp spectrometer can enable significant spatial and spectral-band broadening. Thus, from the starting point design shown in Figure 6b, we investigated how freeform surfaces may be leveraged to simultaneously increase the slit length and the spectral bandwidth over which aberrations are corrected. Each of the three surfaces in the system (that is, the two mirrors and the grating) were made to be freeform and the aberrations were controlled in optimization using the SFFD. Only low-order FRINGE Zernike polynomial surface descriptions up to 21 terms were considered to minimize slope and surface departures. The compact, freeform Offner–Chrisp imaging spectrometer optical performance is given in Figure 7b. The new freeform system is diffraction limited for all wavelengths and fields.

All of the freeform solutions thus far have included freeform terms on the grating surface. For a surface oriented in the *x*-*y* plane, the grating lines are generated by intersecting a series of parallel planes (whose normal vectors point either in the *x* or *y* direction) with the tangent plane of the surface, creating a set of parallel lines. The lines are then translated onto the surface along the local *z* axis, creating the rulings. The technology of ruling a grating on a convex freeform surface has not yet been demonstrated to our knowledge, but is an active research area. PerkinElmer has been ruling gratings on aspherical surfaces successfully for years^34^. The benefit of having freeform contributions at/near the stop is significant, but depending on the application it may not be required. An alternative to having the grating on a freeform substrate would be to manufacture the grating on a spherical convex substrate, then have a nominally flat window located near the convex grating with moderate freeform aberration correction terms polished in. This gives the benefit of having a freeform surface at the stop without the added complexity of manufacturing a grating on a freeform substrate. The cost, however, is the requirement to polish, mount, and align an additional optic in the system.

To further address the potential limitation in fabricating a grating on a freeform substrate, we combined the ideas of a spherical substrate for the grating and having a freeform surface near the stop by choosing a transmissive material for a diffractive catadioptric where the front side (convex) is freeform and the backside (concave) has a reflective grating. This idea is similar to a Mangin mirror^35^. We denote this configuration a ‘Mangin grating’, which is illustrated in Figure 7c. In practice, the grating and the freeform surface of the Mangin grating cannot both be coincident with the aperture stop; instead the grating is set at the stop surface with the freeform surface slightly displaced (by the thickness of the glass or < 5 mm). Because the freeform surface is still close to the stop, its impact on the aberrations is equivalent. The freeform surface on the diffractive catadioptric is used in double pass and, when coupled with a high index material such as zinc sulfide, it can greatly decrease the freeform surface departure for that part when compared to the freeform substrate in Figure 7a. The optical performance for the compact, freeform Offner–Chrisp spectrometer with a Mangin grating is given in Figure 7d.

The advantage of using an all-reflective design is the absence of chromatic aberrations. In the Mangin design, we must address the chromatic aberrations introduced by the refractive element as well as the spectral dispersion at the image plane. After dispersion, each field can be treated monochromatically where each field has a different wavelength compared to its neighbors. There will be a focus error between the fields, yielding essentially a chromatic field-curvature. Likewise, each field will also have a different magnification. This is equivalent to keystone distortion in a spectrometer, which we defined earlier in the paper and will be discussed in the next section. Furthermore, we have constrained in optimization the spectral dispersion in the detector plane to be linear.

***Distortion correction.*** In the various designs reported in this paper, the optical performance (RMS WFE) was stressed as the guiding metric. However, the preliminary aspherical designs resulted in 2 × higher smile/keystone (that is, 0.20/0.40 μm) than the baseline larger volume all-spherical design (that is, 0.10/0.20 μm). When distortion is constrained for the compact aspherical designs, correction comes at the expense of optical performance where the average RMS WFE increased from 0.14 to 5 waves. The freeform design was optimized in two separate cases: once where distortion was unconstrained and another where distortion was softly constrained to <0.1-μm smile and <0.30-μm keystone (that is, <1% and < 3% of a 10-μm pixel, respectively). These values were chosen to match the smile and keystone of the scaled down all-spherical Offner–Chrisp compact design. Distortion for the freeform designs in Figure 7 has been corrected to be less than the constrained values. The distortion corrected designs resulted in different surface departures, however still in the same order of magnitude, but in each case the optical performance was diffraction limited for all wavelengths and fields after optimization. Therefore, distortion correction comes at no penalty to optical performance in the case of the freeform design.

**Figure 8:**
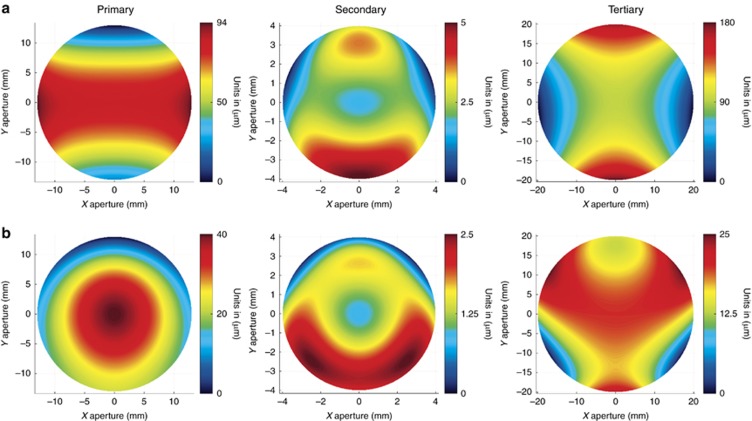
Freeform surface shapes. (**a**) Freeform surface departures from base sphere. (**b**) Freeform surface topography with base sphere and astigmatism removed.

***Manufacturability.*** In the compact freeform Offner–Chrisp imaging spectrometer shown in Figure 7a, the two mirrors and the convex grating are freeform Zernike surfaces. The surface departures from base sphere are shown in Figure 8a. In Figure 8b, we report the departures with sphere and astigmatism removed, which reveals how these surfaces vary from a toroid. The slopes of the surfaces are dominated by the base sphere with an upper bound of 7° of slope over a 13-mm part aperture.

To simulate the as-built performance of one of the freeform Offner–Chrisp spectrometers designed in this work, we applied a root-sum-square (RSS) sensitivity analysis. In this method, the system is individually perturbed with each tolerance. The resulting performance drop is recorded after applying a detector refocus as a default compensator and a tertiary mirror decenter as an additional compensator. The RSS of all performance drops is taken and subsequently added to the nominal performance of the system to yield the predicted as-built performance. The assembly tolerances used here consisted of tip/tilt for each mirror and the detector of ±170 μrad, mirror clocking of ±0.3 mrad, mirror decenters (*X/Y*) and *Z* despace of ±13 μm. The figure error tolerances of each freeform surface consisted of *λ*/4 (*λ*=632.8 nm) PV of Zernike coma, astigmatism and spherical aberration. The compensator refocus was <10 μm and the decenter compensator was <15 μm. The results in Table 2 show that the worst performing point over the image plane corresponding to the maximum RMS WFE is just below the diffraction limit for the chosen tolerances, which are producible by using high-precision machining for the surfaces and the housing, and by adding fiducials and mounting structures on the freeform mirrors during fabrication. Fabrication costs for this first-of-its-kind freeform system would be expected to vary widely depending on the expertise of the chosen manufacturer and the metrology methods used to measure the surfaces.

## Conclusion

In this paper, the benefits of freeform surfaces were revealed in the design of an imaging spectrometer, where the Offner–Chrisp geometry was considered as an exemplary system. In pursuing this design goal, we investigated the impact of various surface types (that is, all-spherical, all-aspherical, freeform) within this equivalent geometry. Performance across all designs was validated and visualized using the SFFD introduced in this paper as a metric to evaluate and visualize the optical aberrations of spectrometers. Freeform surfaces were shown to enable 3 × spectral-band or 2 × spatial broadening when compared to all-spherical and all-aspherical equivalent imaging spectrometer forms. In addition, the freeform imaging spectrometer is shown to simultaneously correct for blurring aberrations and distortion, resulting in superior performance in many applications. Over a fixed spectral bandwidth and slit length it was shown that freeform surfaces decreased the average RMS WFE by 65% compared to both all-spherical and all-aspherical surfaces. Furthermore, combining the concept of broadening the performance in two dimensions, we demonstrated a 5 × reduction in volume using freeform optics in this geometry. We also showed an alternative concept similar to a Mangin mirror where a concave grating was considered as opposed to the classical convex secondary in the Offner geometry. In all freeform designs, performance was shown to be diffraction limited across the visible/near-infrared spectral bandwidth and the slit length.

## Figures and Tables

**Table 1:** Comparison of performance between designs

Designs	All-spherical	All-aspherical	All-aspherical with nodal shift	All-biconic aspherical	Freeform
Average RMS WFE (waves^a^)	0.114	0.107	0.116	0.125	0.040
% Decrease in average RMS WFE compared to all-spherical^b^	NA	6	−1	−9	65
Max RMS WFE (waves)	0.894	0.434	0.254	0.200	0.066
Most departure from best-fit sphere (μm)	NA	0.42	0.20	368	620
Maximum slope departure (°)	3	12	12	7	7

Abbreviations: NA, not applicable.

a Note that as detailed in the SFFD, each point evaluated in the field as a function of the wavelength is normalized to the local wavelength at the point.

b By convention, a minus sign means an increase.

**Table 2:** RSS sensitivity analysis results for an Offner–Chrisp freeform spectrometer

Performance metric	Value (max/min)
Nominal RMS WFE (waves)	0.033/
	0.0093
RSS change (waves)	0.058/
	0.014
As-built RMS WFE (waves)	0.066/
	0.018

RMS WFE is evaluated at the local wavelength of each field point.
